# The Effect of Heat Treatment on the Structure of Zeolite A

**DOI:** 10.3390/ma14164642

**Published:** 2021-08-18

**Authors:** Magdalena Katarzyna Król, Piotr Jeleń

**Affiliations:** Faculty of Materials Science and Ceramic, AGH University of Science and Technology, 30 Mickiewicza Av., 30-059 Krakow, Poland; pjelen@agh.edu.pl

**Keywords:** zeolite A, thermal stability, dehydration, DRIFT spectroscopy, in situ measurement

## Abstract

Knowledge about the thermal properties of zeolites is extremely important due to their potential application in the chemical industry. In this work, the thermal stability and the dehydration process of zeolite A were investigated by in situ high temperature Fourier transform infrared spectroscopy. The progress of thermal decomposition that zeolite A underwent during the controlled temperature increase in the range of 25–600 °C was determined by the DRIFT spectroscopic method. Infrared spectra are presented and discussed for this compound on the basis of the crystal structure. Based on the courses of the obtained DRIFT spectra, it was found that, during heating, water was gradually removed from the structure of the material, followed by dehydration and formation of hydrogen bonds. It was established that the process of thermal degradation began as early as 550 °C. The analysis of the obtained results of structural tests can be repeated on other materials from the zeolite group and complements the research work on the thermal analysis of these materials.

## 1. Introduction

Zeolites are crystalline, hydrated aluminosilicates built of aluminum and silica tetrahedrons (so-called primary building units—PBUs) connected at the corners to form a spatial network [1]. The lattice of these aluminosilicates is composed of one or more structural units which are differently connected, which determines the different zeolite topologies. The zeolite structure is formed by a system of chambers and channels [2]. The chambers are usually in the shape of polyhedra with empty spaces inside. Their size depends largely on the structural arrangement of the PBUs. The size and the shape of the channels are important in selecting cations and molecules that can enter the interior of the crystal.

Due to their properties, zeolites are widely used in various branches of the chemical industry [3]. They can be found in areas such as microelectronics, optics, medicine, and agriculture. The physio-chemical properties that determine such a wide application range include high adsorption capacity, molecular sieve capacity, high selectivity, ion exchange capacity, as well as resistance to acids and elevated temperatures. The high inner surface area of zeolites has many types of active sites of different strength depending on the type of structure. Materials with such a large possibilities of surface and physio-chemical properties modifications are used as adsorbents, ion exchangers, and catalysts.

Among the many known structures, zeolite A deserves special attention. It is one of the most studied and used synthetic zeolites, the discovery of which became a breakthrough for the petroleum processing industry. At the same time, it also became the most commonly used in many areas of everyday life (detergents, insulating glasses, etc.). It is a material obtained by synthesis from readily available raw materials at a not too high temperature, and the process itself is relatively simple [4].

**Figure 1:**
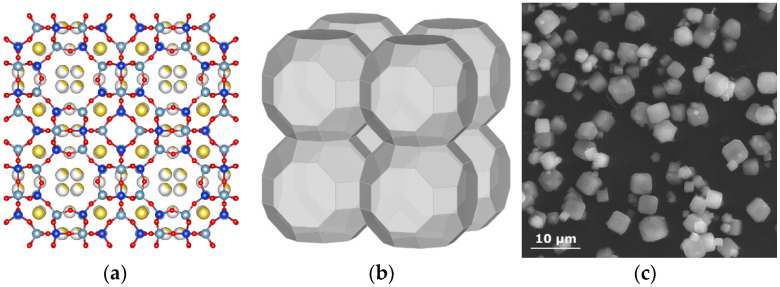
Zeolite Na-A crystal lattice: (**a**) model structure of zeolite Na-A; (**b**) simplified representation of zeolite A crystal structure highlighting the atomic cavities occupied by cations (prepared with the use of VESTA software [10] based on the .cif file available on the [2]); (**c**) SEM image of zeolite A.

Zeolite A was chemically and structurally (both experimental and theoretical) investigated in several early studies [5,6,7,8]. Its ideal chemical formula is Na_96_[Al_96_Si_96_O_384_]·216H_2_O. The zeolite A structure is presented in Figure 1. Its space group was determined to be $\bar{P}3$, and the positions of all non-hydrogen atoms and, thus, the geometry, were provided; positions of the H-bonds were only inferred. Small changes in cation positions and in the zeolite framework were observed upon dehydration [9]. 

Different techniques were applied to analysis of thermal behavior of zeolites. Among other common methods of thermal analysis such as differential thermal and thermogravimetric analysis (DTA and TG), heating in a furnace attached to the diffractometer can be considered as useful. IR spectroscopy is one of the most common methods for investigation of zeolite structure. Using in situ DRIFT technique, the formation of hydroxyl groups, the dehydration/rehydration processes, and the interaction of OH groups with various encaged and adsorbed species can be observed [11]. For example, based on the areas under the DRIFT spectra of zeolite Y, the desorption profiles was calculated [12]. It was found that their shape was principally identical to the desorption curves obtained by conventional temperature-programmed desorption techniques.

Both adsorptive and catalytic properties of zeolites depend not only on the kind of extra-framework cations [13] but also are strongly determined by their water content. Metal impregnation was mainly used to improve the zeolites catalytic properties [14,15]. The aim of this study was to summarize and obtain more detailed structural information on the zeolite A by studying its sodium and ion-exchanged forms. In this study, we examined thermal behavior of zeolite A by in situ high temperature spectroscopic data and discussed the results on the basis of the structural features.

## 2. Materials and Methods

Pure synthetic zeolite A as its sodium form was used as the starting material. Zeolite Na-A was synthesized under hydrothermal conditions using the procedure described in [16]. To obtain other cationic forms, initial zeolite Na-A was treated by water solution of Ca-, Cu-, Ni-, or Zn-nitrates. The concentration of the solutions was 0.01 mol/dm^3^. Ion exchange was carried out at 60 °C for 24 h. After the ion exchange process, the samples were centrifuged and washed several times with distilled water. Phase composition of resulting samples was confirmed using X-ray diffraction. The results were obtained using Empyrean diffractometer (PANanalitycal, Malvern, Great Britain) using Bragg–Brentano geometry and CuKa radiation (measuring time was 4 h, and step over was 0.05°).

The DRIFT measurements were performed on a Vertex 70 v spectrometer (Bruker, Billerica, MA, USA) equipped with a DTGS detector using a Praying Mantis DRIFT attachment (Harrick Scientific Products, Pleasantville, NY, USA). The samples were measured at temperatures up to 600 °C under ambient pressure in Kubelka-Munk scale [17]. Heating rate was set to 5 °C per minute. In total, 64 scans with a 10 kHz scanner velocity were acquired each minute. Spectral deconvolution was carried out using SpectraCalc software (Galactic Industries Corp., Main St Salem, NH, USA). A mixture of Gaussian–Lorentzian with a starting ration of 0.5 was created. As a result, a set of fitted bands with an RMS error below 1 was obtained.

For comparative purposes, the water contents were obtained by heating 10 mg samples in platinum crucibles at 5 °C/min in air using a thermal analyzer Netzsch STA 449 F3 Jupiter (Netzsch, Selb, Deutschland).

## 3. Results and Discussion

**Figure 2:**
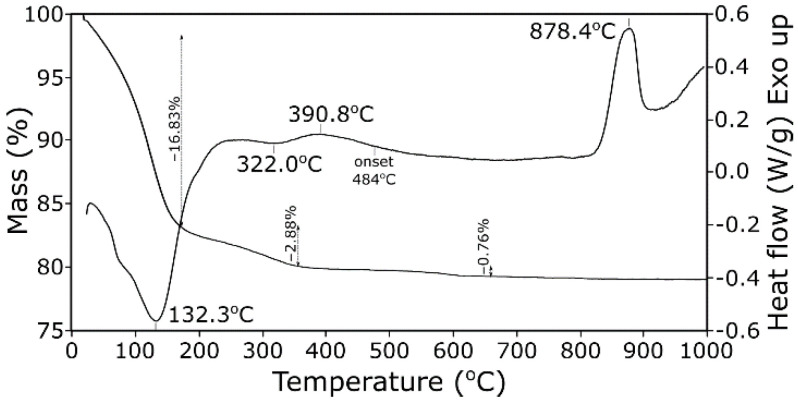
TG-DSC curves of zeolite Na-A.

The average particle size of zeolite A was 1 μm (as observed with SEM; Figure 1c), and it did not contain any other crystalline phases as impurity (checked by XRD; Figure S1). Its dehydration behavior was investigated based on thermal analysis (Figure 2), and selected temperature points were determined for additional structural investigations.

Thermal stability is traditionally determined by thermal analysis. TG-DSC curves of zeolite A are presented in Figure 2. Thermogravimetric analysis showed endothermic weight loss due to dehydration. Mass loss for the quasi-pure zeolite A agreed well with other literature data [1]. At 400 °C, the major part of the dehydration was completed. The sample also contained a small amount of surface and zeolite water, noticeable at a mass loss below 180 °C. The second step with lower slope was observed between 200 and 360 °C (mass loss of 2.9 wt.%). Two step water loss was attributed to subsequent α- and β-cages dehydration [6]. Complete water loss was about 20.5 wt.%, which was slightly less than 22.2 wt.% calculated for Na_96_[Al_96_Si_96_O_384_]·216H_2_O empirical formula) [6]. The obtained value agreed well with the previous results [18,19]. It is also well known [20] that most zeolites are not fully hydrated at room conditions but continue to hydrate as relative humidity is increased to 100%. 

Decarbonation components of the sample could occur from about 300 °C, hence, the presence provided negligible effects on the DSC curve. The zeolite commonly showed an exothermic peak at about 900 °C due to the formation of amorphous aluminosilicate by destruction of the zeolite structure and its subsequent recrystallization into a new phase [21,22]. Dehydrated zeolite A was stable up to 800 °C, when it recrystallized into a β-cristobalite-type structure [1].

**Figure 3:**
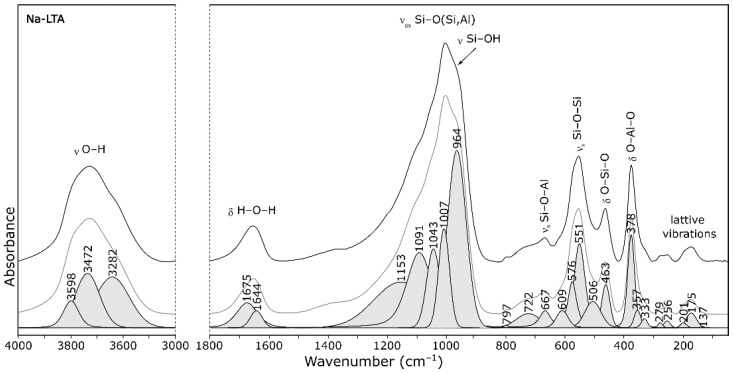
FT-IR spectrum of zeolite Na-A.

The FT-IR spectrum of zeolite A in the 4000–400 cm^−1^ range is displayed in Figure 3 (spectra of all cationic forms are presented as Figure S2). Based on the interpretation of both the theoretical D4R units spectrum [23] as well as the spectrum of the simplified periodic model of zeolite A [24], vibrations of the following bands were assigned:

- 1005 cm^−1^—asymmetric stretching vibrations of bridge bonds—ν_as_ Si–O(Al)
- 726 cm^−1^—symmetric stretching vibrations of bridge bonds—ν_s_ Si–O–Si
- 666 cm^−1^—symmetric stretching vibrations of bridge bonds—ν_s_ Si–O–Al
- 554 cm^−1^—(complex band) symmetric stretching vibrations of bridge bonds—ν_s_ Si–O–Si and bending vibrations—δ O–Si–O
- 465 cm^−1^—bending vibrations—δ O–Si–O, occurring in “antiphase”
- 379 cm^−1^—bending vibrations—δ O–Si–O and δ O–Al–O

Additionally, in the OH stretching region (3000–4000 cm^−1^), very intense peaks in a superposition of several component bands with maximum at ca. 3450 cm^−1^ were observed. Two intense bands were also observed in the H_2_O bending region at ca. 1650 cm^−1^. A very weak and broad band was finally observed around 3245 cm^−1^ that could be finally assigned to the first overtone of the H_2_O bending mode.

**Figure 4:**
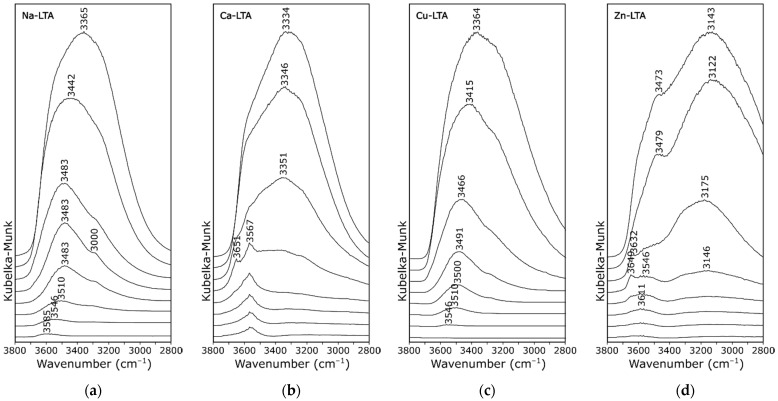
DRIFT spectra of zeolite A recorded at 50 °C intervals (from 50 to 400 °C, respectively, from top to bottom): (**a**) Na-A; (**b**) Ca-A; (**c**) Cu-A; and (**d**) Zn-A.

**Figure 5:**
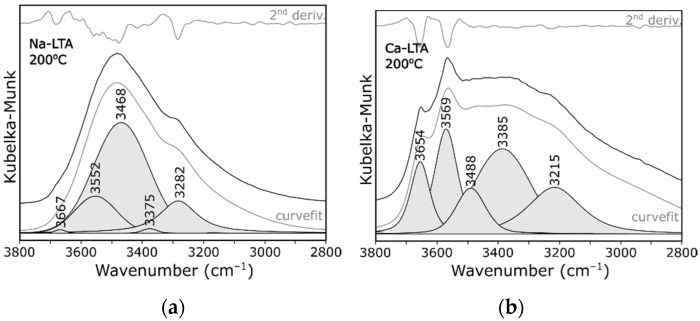
Decomposition of the spectra recorded at 200 °C: (**a**) Na-LTA; and (**b**) Ca-LTA.

**Figure 6:**
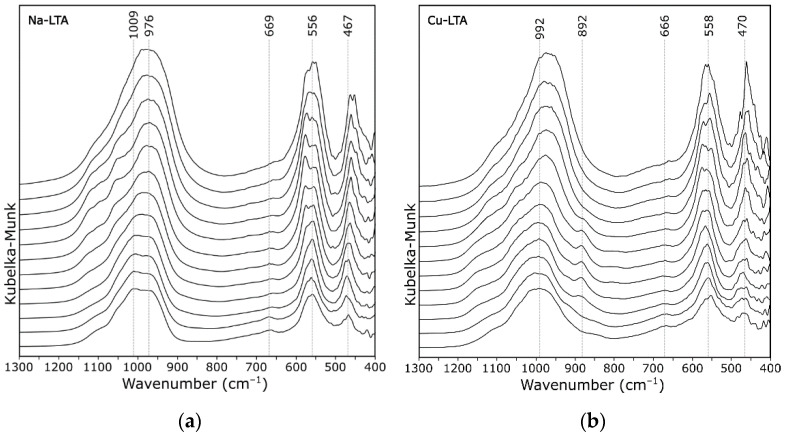
DRIFT spectra of zeolite A recorded at 50 °C intervals (from 50 to 600 °C): (**a**) Na-A; and (**b**) Cu-A.

The DRIFT spectra were recorded as a function of temperature for the sodium form of zeolite A and a zeolite sample exchanged with different metals. Resulting spectra are shown in Supplementary Materials in full wavenumber range as Figures S3–S7. Figure 4, Figure 5, and Figure 6 show the spectra in a smaller wavenumber range.

Changes in the spectral envelope to about 400 °C were mainly related to water loss (Figure 4). The zeolite dehydration took place up to the temperature of about 180 °C, hence, the intensity of the band with a large FWHM (full width at half maximum) disappeared at about 3350 cm^−1^ for all measurement series. At the same time, the band at about 1600 cm^−1^, related to the H−O−H bending vibrations, also disappeared (Supplementary Materials). There is evidence in the literature for the correlation between the integral intensity of the mentioned bands and the amount of adsorbed water, the so-called bulk water [12,25]. This reduction is different for zeolites exchanged with different metals, because metals differ in their ability to surround themselves with water molecules.

The other bands observed in this range were associated with stretching vibrations of OH groups of structural water molecules and OH functional groups. The deconvolution of the exemplary spectra obtained at 200 °C (Figure 5) revealed that, in this range, at least three bands could be distinguished. Two very intense and well resolved peaks at 3468 and 3282 cm^−1^, respectively, were observed in the case of spectrum of zeolite Na-A (Figure 5a). At a closer inspection, an additional two shoulders at 3552 and 3667 cm^−1^ were evident, especially at higher temperatures. These wavenumbers were similar to those described in related literature [26,27]. Following the bands assignment proposed in the work [26], the bands could be assigned successively: 3282 cm^−1^ to molecules arranged in a tetrahedral “ice-like” hydrogen bonded network; 3468 cm^−1^ to water molecules involved in two H-bonds; and 3552 cm^−1^ to molecules of monomeric structures or assembled to form dimers by linear H-bonds. The shifting of the above-mentioned bands towards higher wavenumbers with increasing temperature (Figure 4a) resulted from partial dehydration and, thus, weakening of interactions between individual molecules due to the loss of some hydrogen bonds. The band at about 3580 cm^−1^ disappeared completely at the temperature of 400 °C, which proved complete dehydration and agreed with the results obtained for the TG-DSC analysis (Figure 2). 

The last band (at 3667 cm^−1^) could be assigned to the Si−OH−Al group (bridging hydroxyl groups). This band disappeared above 200 °C. A similar band was attributed to vibrations of corresponding groups in the structure of thermal activated zeolite X [28] or mordenite [29]. By contrast, as the temperature increased, the shoulder at 3650 cm^−1^ was more evident in the case of Ca form (Figure 4b). Similar results were described in the work [30]. Na-A zeolite showed the presence of small amounts of molecular water, while Ca-A zeolite showed the presence of non-acidic CaOH^+^ groups and of symmetrical carbonate ions as well as small traces of acidic OH functionalists. The band at about 3569 cm^−1^ in the spectrum of zeolite Ca-A presented in Figure 4b was caused by Ca(OH)^+^ absorbance. This phenomena can be explained by the Hirschler-Plank dissociative water adsorption model [19]. Interestingly, this band was not observed in any other series of measurements.

It was expected that, with the appearance of stretching vibrations originating from OH groups in the spectrum of the zeolite, the corresponding bending vibrations would also appear. Positions of this band were recognized in the related literature and ranged from 1060 to 1020 cm^−1^ [31,32]. The authors of work [12], on the other hand, noticed that the strong overlapping of the bands due to OH bending vibrations strongly overlapped with the Si−O stretching vibrations and could not be detected with the use of IR investigations. In our work, there were evident bands at about 1055 and 1067 cm^−1^ (Figure 6), which agreed well with the results presented in the work [32].

The copper and the nickel forms of zeolite were selected for this research due to their potential use in catalysis [33]. The envelopes of both spectra (Figure 4c) were similar to the sodium form of zeolite A, possibly because both Cu^2+^ and Ni^2+^ ions occupied the same site as Na^+^ (the center of the six-membered ring in α-cage) [34]. The lack of acid centers could be explained, on the one hand, by the high proportion of aluminum in the zeolite A structure and, on the other hand, the tendency of the analyzed ions to form dimers, e.g., Cu–Cu and Ni–Ni bonds. This property was theoretically confirmed in our previous work [24].

The Zn form of zeolite was characterized by a modified surface charge due to the generation of new Lewis acid sites and variable redox properties of zeolites [35], which influenced, among other things, the selective adsorption of cationic dyes [36]. The zinc form of zeolite A had a completely different course of the IR spectrum in the discussed range (Figure 4d). Some of the zinc ions were probably adsorbed at surface-bridging hydroxyls ≡Si–OH–Al≡ or at surface hydroxyls ≡Si–OH, ≡Al–OH with the formation of Me(OH)(OZe) complex [37]. Hydroxycomplexes Zn(OH)_p_Ze_2_^−p^ Zn, similarly to zinc hydroxides, were more insoluble than the hydroxycomplexes of the remaining investigated metals, which resulted in a large amount of structured water. This hypothesis was confirmed by the appearance of a band at ca. 3649 cm^−1^, analogous to that visible in the spectrum of the Ca-form.

**Figure 7:**
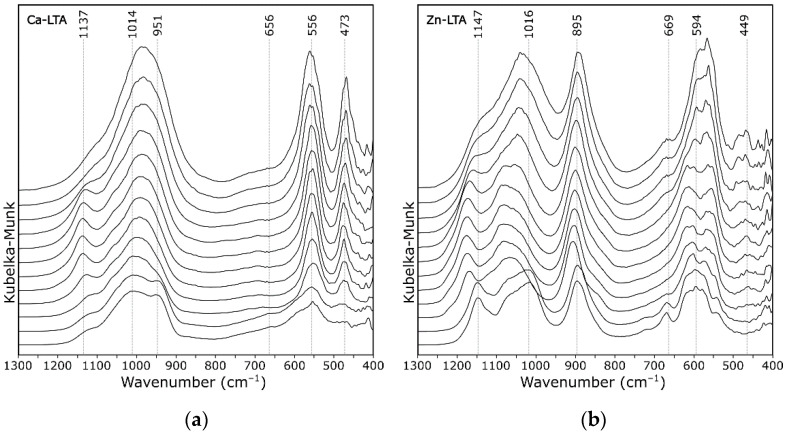
DRIFT spectra of zeolite A recorded at 50 °C intervals (from 50 to 600 °C): (**a**) Ca-A; and (**b**) Zn-A.

Figure 6 and Figure 7 show the DRIFT spectra of zeolite A in the range of Si–O–Al vibration modes. In the initial zeolite (zeolite Na-A; Figure 6a), absorption bands were observed at 1010 cm^−1^ (with a shoulder at 1150 cm^−1^), 950, 669, 556, and 470 cm^−1^. The band at ca. 950 cm^−1^ was due to stretching Si–OH, and its change in intensity corresponded well with the observations for the SiO–H band (at ca. 3600 cm^−1^); it gradually disappeared as the temperature rose. At higher values of the wavenumber, there was a broad band with a maximum at 1010 cm^−1^ and a shoulder at 1150 cm^−1^. Both 1150 and 1010 cm^−1^ bands were attributed to asymmetric stretching vibrations of Si–O. The shoulder was attributed to the location of silicon atoms on different oxygen atoms in the framework. The (Si–O–(Al) band shifted to the lower wavenumber as the temperature increased. The probable reason is that the cation lacking a hydration shell more strongly attracted silicon, which led to an elongation of the Si–O bond (lowering its force constant). The same interpretation could be transferred to other forms of zeolite, e.g., Cu-A in Figure 6b.

The spectra of zeolite Ca-A in Figure 7a, especially Zn-A in Figure 7b, showed a significantly different course compared to the other forms. The differences, however, came down to the shifting of some bands in relation to each other, which gave the visual effect of splitting the bands. Theoretical studies on the structure of the anhydrous form of zeolite A [24] showed that such an effect is a direct consequence of the position of individual extra-framework cations (in relation to the six-membered ring (B site according to [7,38])). The cations that bound more strongly to the skeleton (e.g., d-block cations such as Zn^2+^) distorted it more, resulting in the formation of more than one type of Si–O bond.

According to literature [23,39], the band at about 555 cm^−1^ corresponded to symmetric stretching vibrations of double four-membered rings in the LTA structure. Its complex envelope indicated that it was a superposition of several component bands. Calculated spectra indicated the typical ring vibration (PO—pore opening vibration) gave a band at lower wavenumbers. The presence of this band in discussed spectra indicated that this unit (double four-membered ring) was maintained in the entire analyzed temperature range.

It is well known that zeolite A is not as thermally stable as other zeolites. For example, zeolite Ca-A loses its sorptive ability towards organic vapors if the temperature exceeds 350 °C [40]. At 500 °C, first indications of partial collapse of a zeolite A structure (regardless of the cationic form) were evident in DRIFT spectra (Figure 6). Discussed zeolite structural collapse was followed by formation of an amorphous matter, resulting in an increase in the bands FWHM. It agreed well with the results of X-ray powder diffraction experiments [41] that such a prepared amorphous NaAlSiO_4_ substance was stable between 850 °C and 900 °C. As part of this study, further heating of the system was not possible due to the apparatus limitations resulting from the specificity of the method.

## 4. Conclusions

The DRIFT technique is used to test powders and rough surfaces of various materials. Its unquestionable advantage is the ability to collect spectra in situ conditions, especially useful in the analysis of reactions with the use of catalysts. The technique can be applied successfully in studies of zeolite catalysts.

In this study, we confirmed the course of zeolite A spectra using the DRIFT technique. There were no clear differences in the FT-IR spectra of non-dehydrated selected samples (room temperature) exchanged with different ions. Measurements as a function of temperature allowed a new insight into the zeolite structure. Using DRIFT technique, observations could be made of dehydration and dehydroxylation processes of zeolites as well as some structural changes at higher temperatures.

The essential changes connected with the presence of non-tetrahedral cations could be observed in the expanded spectra in the OH stretching mode region. It was confirmed that, based on this spectral range, three types of water may be recognized in zeolite structure: typical zeolite water, water with crystal-water-like bonds, and water bound to the lattice by OH-bonds.

## Data Availability

Data presented in this study are available on request from the corresponding author.

## Supplementary Materials

The following are available online at https://www.mdpi.com/article/10.3390/ma14164642/s1, Figure S1: XRD patterns of various cationic form of zeolite A, Figure S2: FT-IR spectra of various cationic form of zeolite A, Figure S3: DRIFT spectra of zeolite Na-A recorded at 50 °C intervals (from 50 to 600 °C), Figure S4: DRIFT spectra of zeolite Ca-A recorded at 50 °C intervals (from 50 to 600 °C), Figure S5: DRIFT spectra of zeolite Cu-A recorded at 50 °C intervals (from 50 to 600 °C), Figure S6: DRIFT spectra of zeolite Ni-A recorded at 50 °C intervals (from 50 to 600 °C), Figure S7: DRIFT spectra of zeolite Zn-A recorded at 50 °C intervals (from 50 to 600 °C).
