# Supplementary figures and images for: The Effect of Heat Treatment on the Structure of Zeolite A
Source: Materials (Basel). 2021 Aug 18;14(16):4642. doi: 10.3390/ma14164642 (PMC8401154; doi:10.3390/ma14164642)

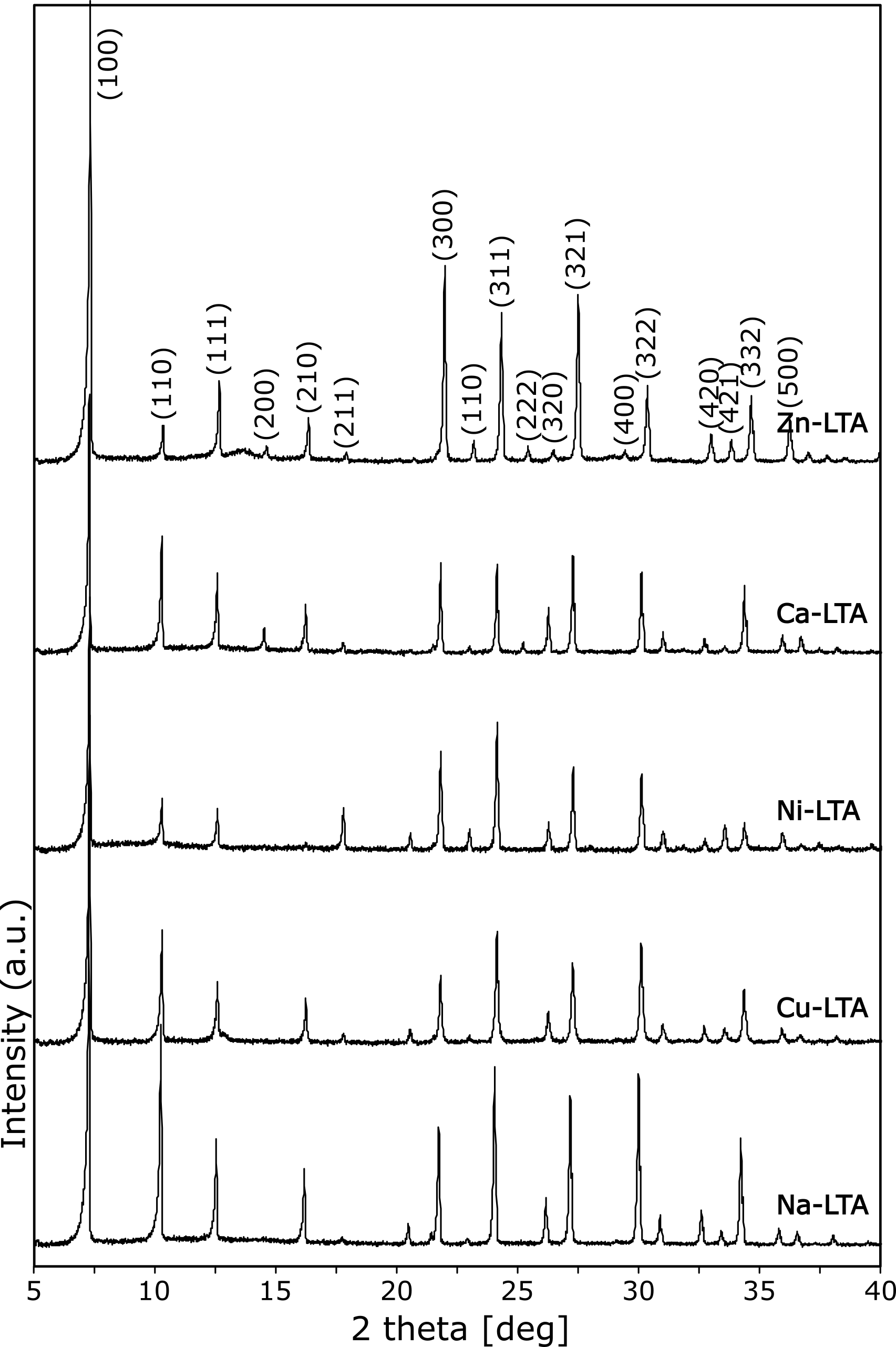

Supplement: Supplementary file 1 [file materials-14-04642-s001.zip › Figure S1.png]

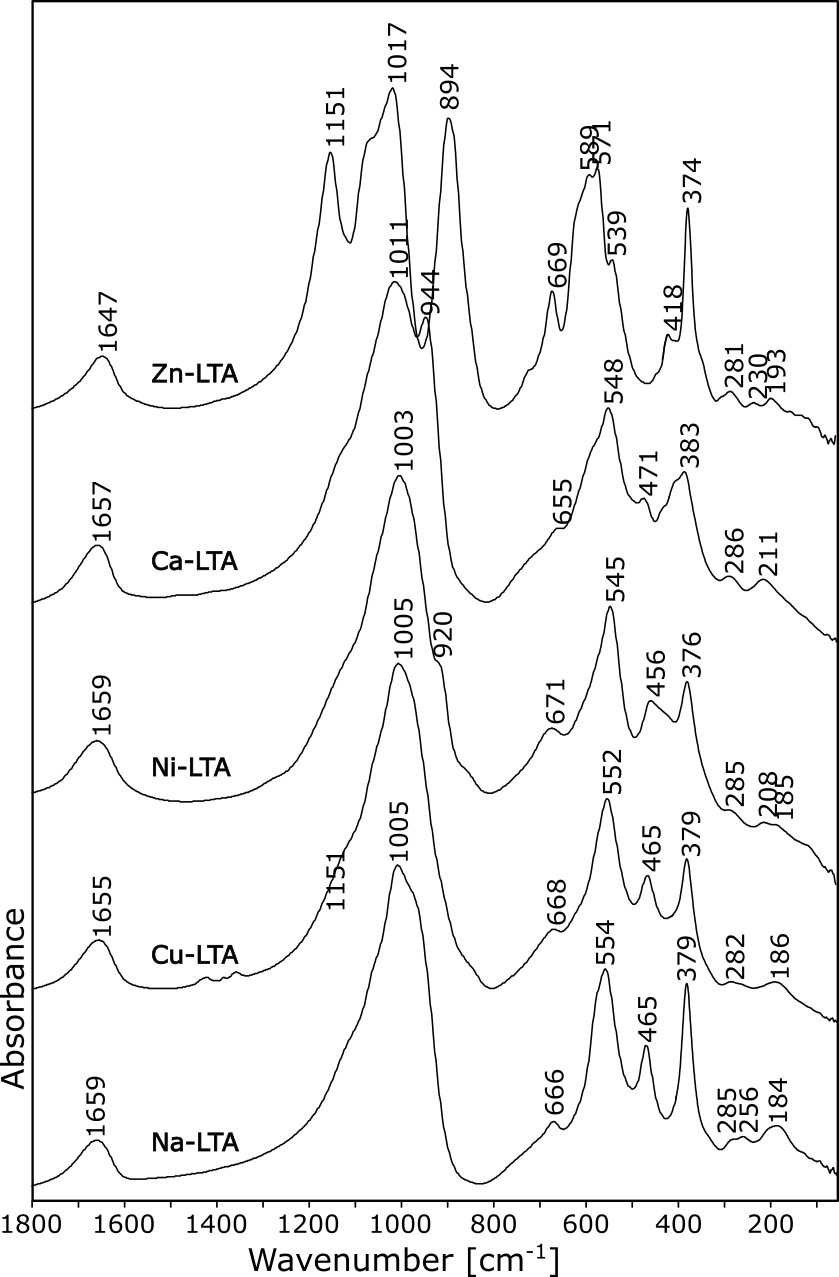

Supplement: Supplementary file 1 [file materials-14-04642-s001.zip › Figure S2.png]

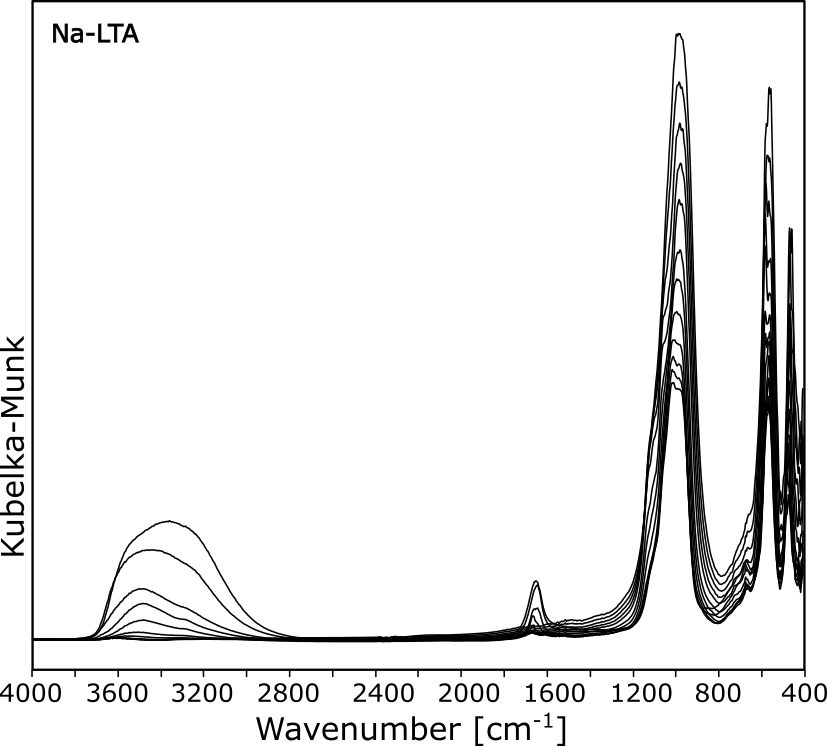

Supplement: Supplementary file 1 [file materials-14-04642-s001.zip › Figure S3.png]

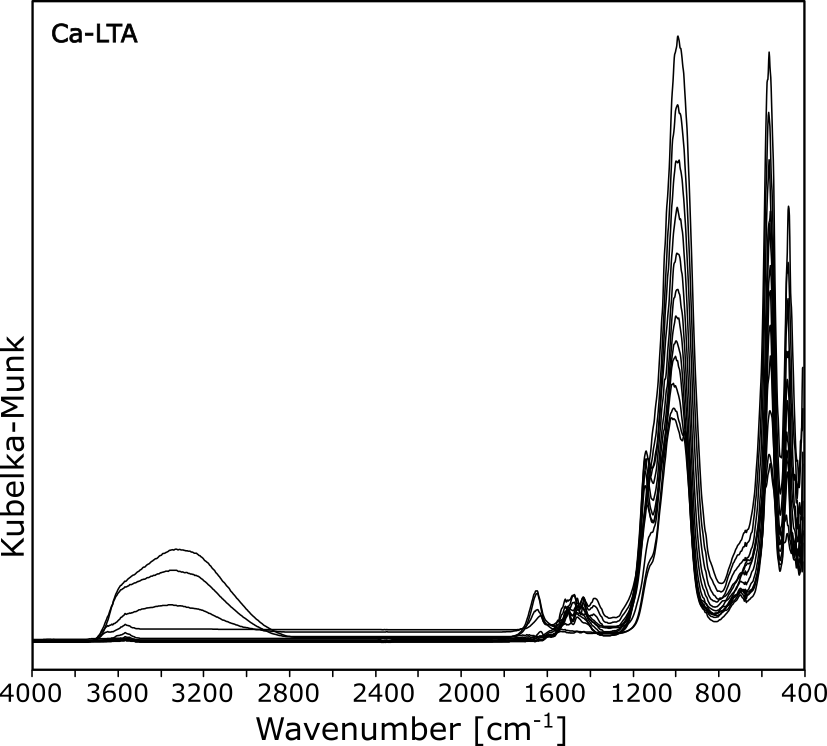

Supplement: Supplementary file 1 [file materials-14-04642-s001.zip › Figure S4.png]

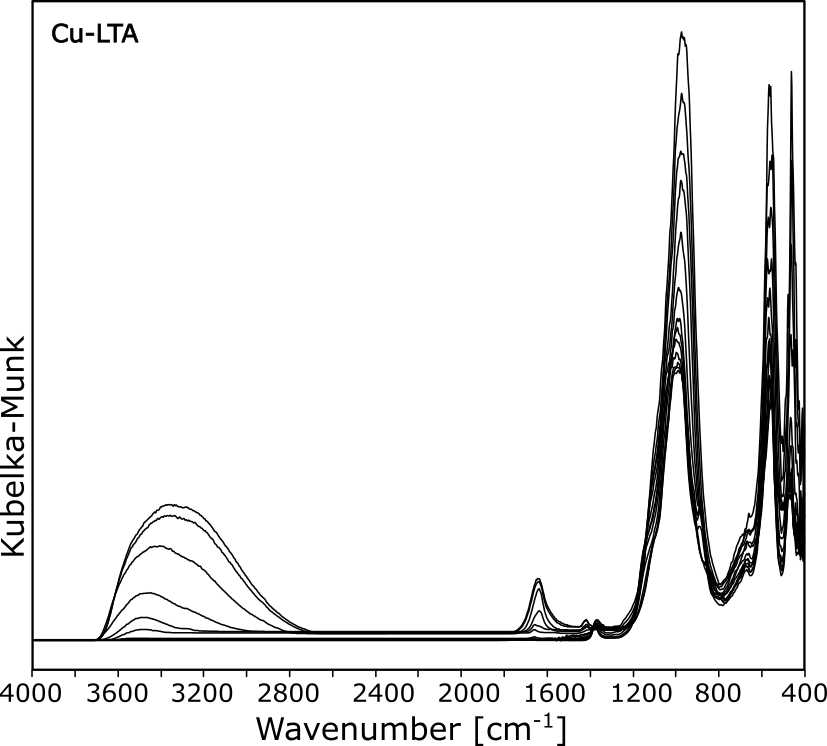

Supplement: Supplementary file 1 [file materials-14-04642-s001.zip › Figure S5.png]

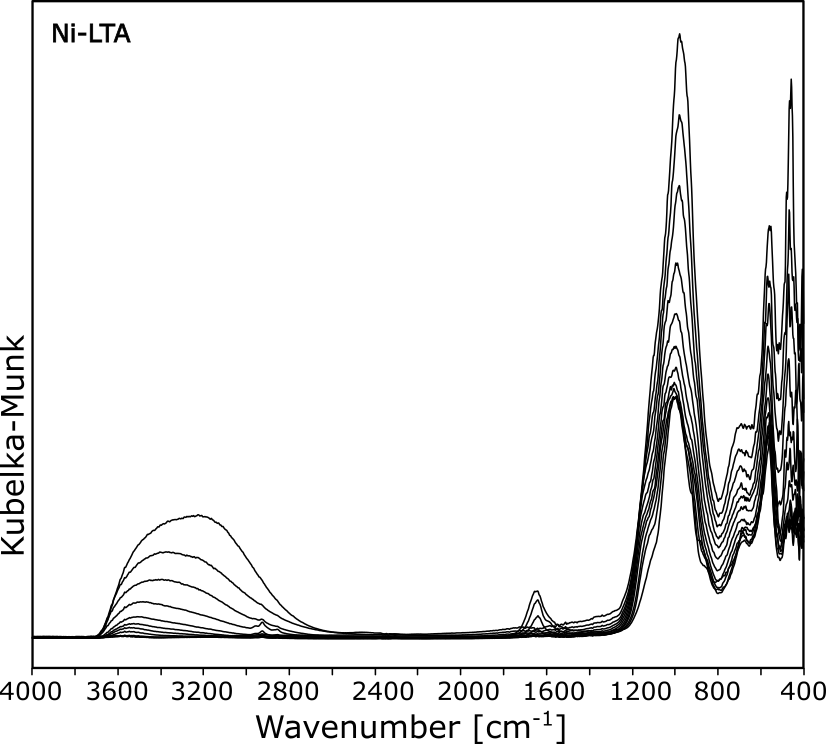

Supplement: Supplementary file 1 [file materials-14-04642-s001.zip › Figure S6.png]

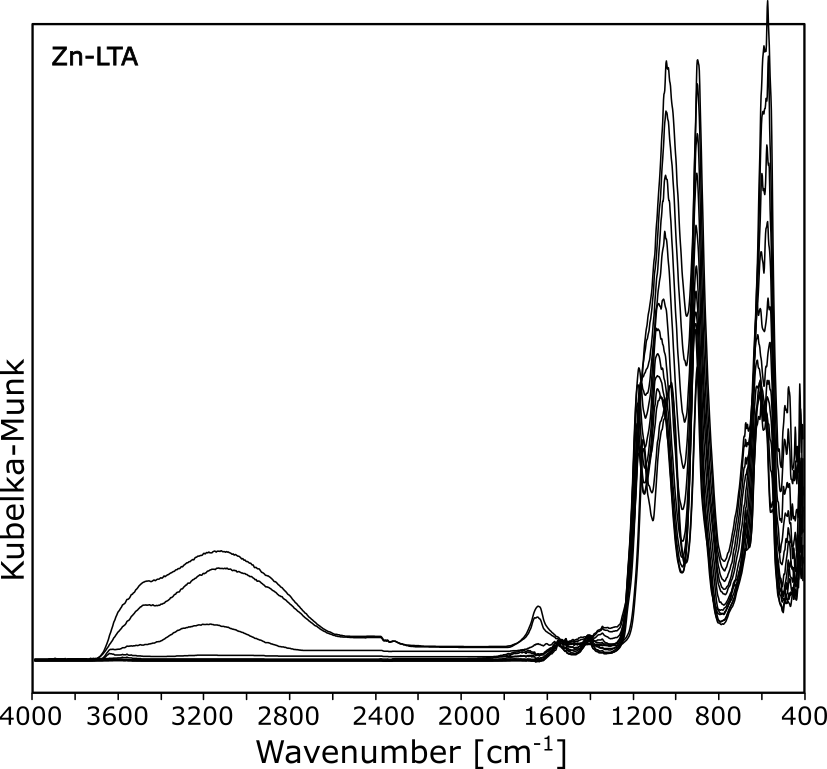

Supplement: Supplementary file 1 [file materials-14-04642-s001.zip › Figure S7.png]
